# Intestinal Barrier in Human Health and Disease

**DOI:** 10.3390/ijerph182312836

**Published:** 2021-12-06

**Authors:** Natalia Di Tommaso, Antonio Gasbarrini, Francesca Romana Ponziani

**Affiliations:** 1 Division of Internal Medicine, Gastroenterology—Hepatology Unit, Fondazione Policlinico Universitario Agostino Gemelli IRCCS, 00168 Rome, Italy; ditommasonatalia@libero.it (N.D.T.); antonio.gasbarrini@unicatt.it (A.G.); 2 Dipartimento di Medicina e Chirurgia Traslazionale, Università Cattolica del Sacro Cuore, Largo F. Vito 1, 00168 Rome, Italy

**Keywords:** intestinal barrier, gut vascular barrier, microbiota, dysbiosis, endotoxemia, gut–brain axis, gut–liver axis, autoimmunity

## Abstract

The intestinal mucosa provides a selective permeable barrier for nutrient absorption and protection from external factors. It consists of epithelial cells, immune cells and their secretions. The gut microbiota participates in regulating the integrity and function of the intestinal barrier in a homeostatic balance. Pathogens, xenobiotics and food can disrupt the intestinal barrier, promoting systemic inflammation and tissue damage. Genetic and immune factors predispose individuals to gut barrier dysfunction, and changes in the composition and function of the gut microbiota are central to this process. The progressive identification of these changes has led to the development of the concept of ‘leaky gut syndrome’ and ‘gut dysbiosis’, which underlie the relationship between intestinal barrier impairment, metabolic diseases and autoimmunity. Understanding the mechanisms underlying this process is an intriguing subject of research for the diagnosis and treatment of various intestinal and extraintestinal diseases.

## 1. Introduction

The gastrointestinal mucosa is the surface where the interactions between humans and the external world take place. It is composed of multiple layers, each of them with a specific role, as the integrity of the intestinal barrier is necessary for our sustenance, health and defense [1].

The human gut is also inhabited by a huge community of microorganisms, including bacteria, viruses, fungi and helminths, that are all included under the definition of “gut microbiota” [2]. The genetic material of these microbes, the “gut microbiome”, consists of more than 45 million non-redundant genes, when only the oral and gastrointestinal microbiome is considered [3], and provides for a number of functions that integrate and complement that of the human genome. The gut microbiota and the intestinal barrier communicate with each other, realizing a complex network of interactions that, in physiological conditions, are in balance, contributing to human body homeostasis and health. 

Perturbations deriving from foods, physical conditions, and chemical substances, as well as from modifications of the gut microbiota composition and function, can potentially alter this equilibrium. Therefore, modulation of the gut microbiota–intestinal barrier interactions is increasingly being considered as a target of new therapeutic strategies in several intestinal and extraintestinal diseases [4].

## 2. Composition of the Intestinal Barrier

The intestinal barrier is composed of multiple layers. The outer one comprises the mucus layer, the commensal gut microbiota and defense proteins such as antimicrobial proteins (AMPs) and secretory immunoglobulin A (sIgA). Intestinal epithelial cells (IECs) are the middle layer, while the inner part is composed of immune cells of innate and adaptive immunity [5].

### 2.1. Mucus Layer

The mucosal surface of the gastrointestinal tract is covered by mucus, a substance composed mainly of water (normally >98%) and the proteins MUC2 and MUCA5C in the stomach, which are produced by goblet cells [6]. In the stomach and colon there are two layers of mucus, whereas in the small intestine there is only one layer [7]. The microbiota residing in the intestinal mucosa colonizes the outer layer of mucus in the large intestine, without making contact with the epithelium, whereas in the small intestine this contact occurs only at the tips of the villi [6]. Because in the small intestine the mucus layer is thinner and more penetrable by bacteria or potential toxins, enterocytes, Paneth cells, and immune cells secrete antimicrobial proteins for host defense [8]. 

The role of the mucus layer is to protect intestinal cells from external agents and to facilitate nutrient absorption [9]. Several factors contribute to mucus metabolism. The microbiota can regulate the intestinal environment and influence the integrity and function of the outer mucus layer, because germ-free animals have a thinner mucus layer and fewer goblet cells [10]. In this model, bacterial products such as lipopolysaccharide (LPS) and peptidoglycan stimulate mucus secretion and restore mucus properties [11]. At the same time, MUC2-deficient mice are more susceptible to colitis [12]. In contrast, some resident bacteria such as *Akkermansia muciniphila*, *Bacteroides thetaiotaomicron*, *Bifidobacterium bifidium*, *Bacteroides fragilis*, and *Ruminoccous gnavus* degrade mucus for their own metabolism and that of other commensals in a homeostatic balance [13]. This process is enhanced on a low-fiber diet, as fiber represents an energy source for the microbiota [14]. Immune cells also regulate mucus metabolism through cytokine secretion [15]. For example, IL-4 has been shown to increase mucus thickness in a mouse model of *Citrobacter rodentium* colitis [16], whereas overexpression of IL-18 has been associated with goblet cell downregulation [17]. 

### 2.2. Epithelial Cells

Five distinct types of cells compose the epithelium of the intestinal barrier. These are enterocytes, goblet cells, enteroendocrine cells, Paneth cells and microfold cells. These cells are renewed by a pool of stem cells residing in the intestinal crypts [5].

The gut epithelium is impermeable to hydrophilic solutes, so molecules and nutrients can only pass through it via specific transporters. There are two main pathways: the transcellular route, including aqueous pores, active carrier-mediated absorption for nutrients and endocytosis; and the paracellular route, for ions and hydrophilic molecules. The paracellular pathway is regulated by junctional complexes, a group of proteins consisting of tight junctions (TJs), adherens junctions, desmosomes, and gap junctions [18]. TJs play a key role in the integrity of the intestinal barrier. They are composed of three groups of transmembrane proteins (claudin family, the Marvel domain-containing proteins, and immunoglobulin superfamily), which interact with the cytoskeletal actomyosin ring [19].

Under conditions of homeostasis, TJs help select the passage of substances through two pathways, the “pore” pathway, which is highly selective, and the “leak” pathway, which has limited selectivity. Therefore, TJs represent a mechanical division between the luminal space and the other components of the intestinal barrier [20].

### 2.3. The Gut Microbiota

The gut microbiota is composed of 100 trillion microorganisms [21]. It is involved in protective, metabolic, and structural functions for the intestinal tract and host. Its composition and function can change with age and different health conditions, resulting in changes in the structure and function of the intestinal barrier as well. 

One of the main functions of the gut microbiota is to secure nutrients to intestinal cells and metabolize undigested products of diet such as protein and dietary fiber [4]. Specifically, through anaerobic fermentation of undigested complex carbohydrates, the gut microbiota can generate short-chain fatty acids (SCFAs). SCFAs consist of butyric, propionic and acetic acids, which are not only an energy substrate for intestinal epithelial cells but are also implicated in regulatory functions. Other SCFAs such as formate, valerate, and branched-chain fatty acids deriving from amino acid catabolism have minor implications in gut homeostasis [22]. 

SCFAs can inhibit histone deacetylases (HDACCs) and bind G protein-coupled receptors (GPCRs) promoting the expansion of hematopoietic and nonhematopoietic cells. cells. They reduce cytokine production by neutrophils and macrophages, inducing an immunotolerogenic phenotype [23,24,25]. SCFAs can also increase mucus layer production by modulating the transcription of mucin genes in goblet cells [26]. SCFAs, particularly sodium butyrate, can promote TJ reassembly by regulating AMP-activated protein kinase (AMPK) activation and phosphorylation of myosin II regulatory light chain (MLC2), reinforcing the intestinal epithelial barrier [27,28].

Finally, the gut microbiota aids the development of the host immune system through metabolites, microorganism-associated molecular patterns (MAMPs), including pathogen-associated molecular patterns (PAMPs), and antigens [29]. Bacterial translocation is a process defined as the migration of pathogens, or their products, from intestinal lumen to mesenteric lymph nodes [30]; lipopolysaccharide (LPS), a component of the wall of Gram-negative bacteria, is one of the MAMPs recognized by receptors on cells of the innate immune system, such as Toll-like receptors (TLRs) [31]. TLRs are a family of pattern recognition receptors (PRRs) normally expressed on the surface of intestinal and immune cells as transmembrane proteins and capable of recognizing bacterial, viral or parasitic ligands [32]. Their activation causes the induction of the nuclear transcription factor-kappa B (NF-kB), a group of transcription factors involved in the production of inflammatory cytokines [33]. TLRs trigger immune response through NF-kB when stimulated by pathogens, but they can also be activated by commensal bacteria. This symbiotic interaction plays a protective role in intestinal homeostasis; in fact, mice deprived of Myd88, a protein activated by the TLR pathway, or those deprived of the microbiota by antibiotic cocktails are more prone to develop severe colitis after administration of dextran sulfate sodium (DSS) [34,35]. In addition, some microbial species such as Bacteroides fragilis can promote Foxp3+ regulatory T-cell expansion and IL-10 secretion through the expression of polysaccharide A (PSA), a capsular carbohydrate recognized for its immunomodulatory and anti-inflammatory properties in experimental models of colitis [36,37,38].

### 2.4. Immune Cells

Intestinal immunity is provided at the mucosal surface by intestinal epithelial cells, intraepithelial lymphocytes, and, in the small intestine, Paneth cells. In addition, the lamina propria is colonized by cells of innate and adaptive immunity [39], and gut-associated lymphoid tissue (GALT) can be found in the small intestine as large aggregates of lymphoid tissue called Peyer’s patches, and as isolated lymphoid follicles in both the upper intestine and colon [40]. Gut cells and Paneth cells represent the first line of defense against pathogens and contribute to host immunity through the secretion of cytokines and antimicrobial products, such as defensins [41]. Paneth cells play an antimicrobial and microbiota-modulating role through the secretion of lysozyme, defensin, and other immunoregulatory proteins; in particular, among other cytokines, IL-22 has been recognized to have multiple roles in intestinal health [42,43]. Indeed, IL-22 is involved in epithelial cell regeneration, mucus homeostasis through the induction of mucin genes and goblet cells, and the release of antimicrobial peptides. Paneth cells and IL-22 dysregulation have been linked to the pathogenesis of Crohn’s disease by promoting intestinal inflammation [43,44].

**Figure 1:**
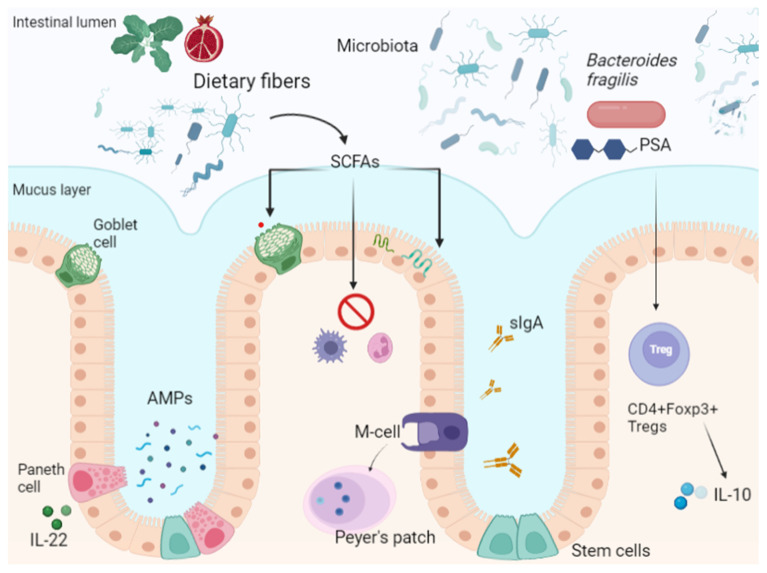
Intestinal barrier composition. SCFAs: Short chain fatty acids; M-Cells: Microfold cells; PSA: Polysaccharide A; AMPs: antimicrobial peptides; sIgA: secretory IgA.

Peyer’s patches are the sites of induction of IgA positive plasma cells in response to pathogens and immune cell signaling [45]. Immunoglobulin A is the first serum immunoglobulin, participates in mucosal immunity [46], and regulates inflammatory responses against commensal bacteria, avoiding dysbiosis [47,48]. As emerged from an experimental study on mice, IgA coating is also a mechanism of recognition of possible colitogenic bacteria from the gut microbiota [49]. Finally, microfold cells are specialized epithelial cells that participate in inflammatory responses by capturing antigens in the luminal surface of the intestinal mucosa and transporting them to Peyer’s patches through transcytosis [50]. Their activity is required for the modulation of secretory IgA [51], and experimental data demonstrate that they are able to reduce bacterial translocation [52] (Figure 1).

SCFAs produced by the microbiota’s metabolism of dietary fiber regulate the mucus layer produced by Paneth cells, modulate inflammatory cytokine production, and strengthen epithelial tight junctions, contributing to intestinal homeostasis.

Paneth cells are a subtype of specialized epithelial cells located in the small intestinal crypts; they contribute to host immunity through secretion of antimicrobial products (AMPs). IL-22 can also stimulate AMP secretion by Paneth cells.

Intestinal M cells participate in mucosal immunological surveillance and induce IgA-secreting plasma cells. Secretory IgA colonizes the mucus layer and modulates microbiota–host interactions. 

The gut microbiota and its fragments (microorganism-associated molecular patterns (MAMPs) and pathogen-associated molecular patterns (PAMPs)) and products physiologically translocate across the intestinal barrier, and are recognized by specific receptors (toll-like receptors, TLRs) on immune cells, maintaining immune homeostasis and self-tolerance.

### 2.5. External Factors

Among the environmental factors that can affect gut health, air and particulate pollution, pesticides, food additives, and xenobiotics are known modulators of the gut barrier. 

Experimental models on Caco-2 cells show that pollution can induce oxidative stress in intestinal cells through disorganization of TJs, increasing intestinal permeability [53]. 

Cigarette smoking is an important risk factor for cancer [54]. Smoking also has several effects on the composition of the gut microbiota, which can be reversed after smoking cessation. A cross-sectional study reported higher fecal abundance of Bacteroidetes in current smokers compared to never and former smokers [55]; moreover, smoking alters oral microbiota composition, predisposing to a pathogen-rich microenvironment associated with periodontal diseases [56]. In mice, smoke effects on villi and TJs integrity were associated with increased small intestine permeability [57] Nicotine plays a protective role in ulcerative colitis (UC) by modulating cholinergic pathways in immune cells and reducing their activation and cytokine expression [58], while in Chron’s disease (CD) it increases intestinal permeability by interacting with claudin gene expression [59], leading to apoptosis in the follicle-associated epithelium [60] and change in the composition of the gut microbiota [61].

Studies in rats have shown that pesticides such as chlorpyrifos, an organophosphate insecticide, increase intestinal permeability [62] and are able to dysregulate the expression of TJ proteins such as zonulin-1 (ZO1) and claudin-4 [63]. Glyphosate, an organic acid used as a herbicide, can affect the composition of the gut microbiota by interfering with bacterial biosynthesis of aromatic amino acids, with uncertain effects in in vivo studies [64]; it can also induce membrane damage in Caco-2 cells and rat intestinal cells at high doses [65].

Food additives, including natural ones, are represented by antimicrobials, antioxidants, and sweeteners used to ensure preservation of food products [66]. Recently it has been postulated that food additives could increase the susceptibility to intestinal autoimmune diseases by exerting direct effects on TJs [67]. For example, some authors explain the increasing incidence of CD in Japan by citing the extensive use of emulsifiers in this country [68].

During heat stress, the intestinal barrier is compromised and becomes permeable to macromolecular proteins, including toxins, antigens, and potential allergens [69]. The proposed mechanism involves the activation of protein kinase C (PKC), which in turn phosphorylates regulatory light chain of myosin II (MLCK), leading to TJ derangement through increased actin contractility [70]. Studies of athletes during high-intensity exercise confirm that metabolic heat production and changes in blood supply to the intestinal mucosa affect the integrity of the intestinal barrier [71,72].

Finally, xenobiotics such as drugs can alter the integrity of the intestinal barrier. Among the most widely used drugs, proton pump inhibitors (PPIs) are important modifiers of the gut microbiota composition, mainly through the increase in gastric pH. The impairment of the acid barrier exposes the intestinal tract to infections with various microorganisms [73] and modifies the gut microbiota, reducing its diversity and promoting the proliferation of oral bacteria in the small intestine [74]. This process is called “gut oralization”, and is associated with bacterial translocation and liver damage in alcoholic and metabolic liver diseases [75]. In addition, the increase in gastric pH mediated by these drugs is recognized as a risk factor for *Clostridium difficile* infection [76]. PPIs can also modulate the intestinal barrier, interfering with smooth muscle relaxation, phosphatase activity, and local electrolyte homeostasis (i.e., Ca and K), and hence, with the function of TJs [77,78,79]. Nonsteroidal anti-inflammatory drugs (NSAIDs) are also associated with a specific form of enteropathy represented by mucosal damage and ulceration visible on endoscopy [80]. The pathogenesis is unclear, but probably related to mitochondrial membrane function and ATP production. Indeed, dinitrophenol, an uncoupling agent, was able to increment intestinal permeability in a mouse model of NSAIDs enteropathy when co-administered with indomethacin [81,82].

## 3. The Intestinal Barrier in Autoimmune, Metabolic, and Neurological Diseases

In cases of severe mucosal damage, intestinal permeability increases, and bacteria, their fragments, or products can translocate through the lumen. Interaction between LPS and TLRs leads to systemic endotoxemia [83,84]. The hypothesis that an altered intestinal barrier may lead to an increased intestinal permeability and inflammatory response, and that the gut microbiota may modulate this process, has led to the concept that “leaky gut syndrome” and “dysbiosis” are linked to each other, and that both are involved in the pathogenesis of various gastrointestinal and systemic disorders [85,86]. Indeed, intestinal barrier dysfunction has been associated with various diseases, from autoimmune (inflammatory bowel diseases [IBDs], type 1 diabetes mellitus, celiac disease, multiple sclerosis, etc.) [87] to neurological ones (mood disorders, autism spectrum disorders, Parkinson’s disease, Alzheimer’s disease) [88,89,90], playing the role of a primer or aggravating factor in their evolution.

### 3.1. Inflammatory Bowel Diseases

Inflammatory bowel diseases (IBDs), such as CD and UC, are chronic disorders, and their exact etiology is still unknown. Immunological, genetic, and environmental factors and changes in the gut microbiota are the most likely factors involved in the pathogenic mechanism [91].

Intestinal leakiness in patients with IBDs is related to dysbiosis, inflammatory response, and TJs modifications. The gut microbiota of patients with IBDs is characterized by an increase in pro-inflammatory bacteria, such as adherent–invasive *Escherichia coli* or mucolytic bacteria such as *Ruminococcus gnavus* and *Ruminococcus torques*, and a reduction in the gut microbiota diversity [92]. A major variability in the gut microbiota composition has also been reported, together with a lower abundance of *Subdoligranulum* species, the clinical relevance of which is under investigation [93]. During active IBDs, the expression of enterotoxigenic *Bacteroides fragilis*, a metalloprotease-producing bacteria, is increased, causing inflammatory diarrhea [94]. Altered expression of tumor necrosis factor-alpha (TNF-*α*), transforming growth factor-beta (TGF-*β*), interleukin- (IL-) 17, IL-22, and IL-23, and mutations in the NOD gene, a member of PRRs family, are involved in the pathogenesis of IBDs, resulting in an enhanced inflammatory response in the intestinal mucosa [95]. In CD, mutations in NOD2 cause altered expression of defensin genes, facilitating an altered antimicrobial response to the gut microbiota and translocation of bacteria across the epithelium [96]. Multi-omics studies on IBD patients also showed a metabolic reduction in SCFAs and an increase in polyunsaturated fatty acids, including arachidonate, among dysbiotic patients, and a reduction of bile acids (BAs) conversion, with an overall pro-inflammatory effect [93]. 

Dysregulation of TJ complexes has also been demonstrated. Indeed, TNF-*α* is implicated in TJ modifications, and together with IL-13, promotes intestinal cell apoptosis in UC [97,98].

Interestingly, altered intestinal permeability can be exacerbated during IBDs by a variety of external factors, but it has been shown to occur in asymptomatic patients years before the onset of clinical manifestations [99]. In a study of 1420 first-degree relatives of CD patients, intestinal permeability was measured in vivo using urinary fractional excretion of lactulose/mannitol ratio (LMR). The study showed that LMR expression predicts the onset of CD by years, representing a possibly preclinical marker of the disease and paving the way for new target therapies [100].

### 3.2. Rheumatoid Arthritis

Experimental models of rheumatoid arthritis (RA) (collagen-induced arthritis-CIA) showed that the genus Lactobacillus was overrepresented in CIA- susceptible mice before the onset of arthritis [101]; at the same time, *Lactobacillus salivarius* is overabundant in the oral microbiota of individuals with active RA [102]. Jubair WK et al. showed that gut dysbiosis in CIA-predisposed mice was correlated with the degree of inflammation and permeability of the intestinal mucosa, with increased intestinal expression of IL-17A and IL-22 [103]. Furthermore, fecal microbiota transplantation (FMT) from CIA mice to germ-free mice is associated with the development of RA [101]. These data suggest the existence of a mucosal susceptibility for the development of RA, the so-called gut–joint axis, but further evidence is needed for possible therapeutic challenges [104].

### 3.3. Ankylosing Spondylitis

Patients with ankylosing spondylitis (AS), a form of spondyloarthritis, show subclinical intestinal inflammation, which sometimes evolves into IBD [105], and a higher gut microbiota diversity compared to healthy subjects [106]. A study including patients with AS and CD and healthy controls demonstrated that both AS and CD patients had high IL-23 mRNA expression in mucosal biopsy specimens. Two IL-23 expression pathways were observed: the first was associated with Il-23 production by immune cells infiltrating the intestinal mucosa; in the second case, IL23 was produced by Paneth cells in intestinal crypts [107]. Paneth cell activation in response to intestinal dysbiosis and the consequent release of cytokines in the systemic circulation could be responsible for early manifestations of AS, supporting the existence of a gut–joint axis [108].

### 3.4. Systemic Lupus Erythematosus

Patients affected by systemic lupus erythematosus (SLE) are colonized by a less heterogeneous gut microbiota, with increased abundance of Gram-negative bacteria [109]. 

In experimental models, after DSS administration, SLE-predisposed mice showed an elevated inflammatory response secondary to increased intestinal permeability, with production of autoantibodies and the spread of systemic inflammation [110]. 

Interestingly, FMT from SLE-affected mice into germ-free mice is associated with an incremental immune response in the intestinal mucosa and production of anti-dsDNA antibodies, suggesting a possible pathogenic link between dysbiosis and SLE [111].

### 3.5. Parkinson’s Disease

Patients affected by Parkinson’s disease (PD) often complain of gastrointestinal symptoms years before the diagnosis [112], and accumulation of α-synuclein was observed in submucosal colonic neurites of early diagnosed or untreated patients [113]. In addition, recent findings support the hypothesis that a-synuclein is formed in the gut and then transferred to the central nervous system via the vagus nerve [114].

Recently, an experimental study in mice analyzed the relationship between stress, intestinal permeability, and neuroinflammation [115]. Administration of rotenone, a pesticide used to induce PD in rats [116], together with restraint stress, led to increased intestinal permeability with an additive effect. Indeed, rotenone and restraint stress were able to destroy TJ proteins in the intestinal mucosa and increase plasma LPS levels. At the same time, tissue analysis of treated mice showed high levels of α-synuclein in myenteric plexuses and signs of microglial neuroinflammation in the substantia nigra. This study explains a possible relationship between stress, leaky gut, and the development of PD [115]. 

### 3.6. Autism Spectrum Disorders

Children with autism spectrum disorders (ASD) often experience intestinal symptoms, such as constipation, abdominal pain and diarrhea [117]. Microbiota studies in these patients have shown an inverse relationship between microbiota diversity and neurological impairment [118], and a modification of Firmicutes/Bacteroidetes ratio, caused by a reduction of *Bacteroidetes* bacteria [119].

Neuroinflammation is a recognized pattern in ASD [120]. A human study conducted postmortem in patients affected by ASD and schizophrenia showed increased expression of two markers of neuroinflammation and blood–brain barrier impairment, matrix metallopeptidase 9 (MMP9) and 18 kDa translocator protein (TSPO), in the cerebral cortex [121]. In addition, reduced expression of TJ proteins was observed in the intestinal mucosa. This study could explain a possible pathological link between intestinal disorders and the development of autism [122].

### 3.7. Type 1 Diabetes

Enterovirus infections in early life represent a predisposing factor for the development of autoantibodies associated with type 1 diabetes mellitus (T1DM), probably triggering gut mucosal barrier damage [123]. Alteration of TJs has been proposed as the hallmark of intestinal dysfunction in diabetes. Studies have shown that alterations in intestinal permeability occur before the onset of T1DM. In a rat model of T1DM, luminal and serum levels of ZO1 were higher in diabetic mice than controls, and preservation of TJ integrity by pharmacological inhibition of the ZO1 receptor was able to reduce the risk of T1DM development in predisposed mice [124,125]. 

T1DM-associated dysbiosis, particularly the lack of butyrate-producing bacteria, can further contribute to the alteration of intestinal permeability in this setting, as butyrate stimulates mucin secretion and helps maintain the integrity of TJs [126].

### 3.8. Type 2 Diabetes

In patients with type 2 diabetes mellitus (T2DM), hyperglycemia promotes a pro-inflammatory condition that is related to intestinal permeability, bacterial translocation, and metabolic endotoxemia [127,128]. Indeed, chronic hyperglycemia drives intestinal barrier dysregulation by direct action on gene transcription [129]. 

Microbiota changes in T2DM involve decreased abundance of *Bifidobacterium*, *Bacteroides*, *Faecalibacterium*, *Akkermansia*, and *Roseburia*, with metabolic consequences [130]. Bacteroides acidifaciens is positively associated with increased insulin sensitivity in peripheral tissues [131], while the lack of *Akkermansia* is directly associated with the risk of metabolic syndrome [132]. The benefits of Akkermansia on glucose metabolism have recently been linked to GLP-1 secretion [133]. Interestingly, in experimental models, metformin increases Akkermansia expression in the gut [134]. 

Changes in the gut microbiota composition in T2DM patients are also associated with reduced production of SCFAs from dietary components, adversely affecting intestinal barrier function, regulation of inflammation, and lymphocyte function [135]. This condition promotes a pro-inflammatory pathway that increases metabolic endotoxemia and oxidative stress [136], which in turn enhance insulin resistance and beta-cell impairment [137]. 

### 3.9. Obesity

Obesity is often associated with metabolic syndrome and insulin resistance [138]. Chronic inflammation, driven by the pro-inflammatory activity of macrophages in the adipose tissue, colon, muscle, and liver, is the substrate for these conditions, and is called “metainflammation” [139]. Gut dysbiosis in obesity is associated with a high Firmicutes/Bacteroidetes ratio in most studies [140] and with an increase in potentially pro-inflammatory and invasive bacteria [141]. 

A high fat diet (HFD) promotes gut leakiness through dysbiosis, since antibiotic treatment has been shown to be effective in improving intestinal permeability and glucose homeostasis in HFD-fed mice [128]. However, in an experimental model of leptin-deficient mice, obesity per se was associated with increased intestinal permeability, due to reduced expression of occludin, ZO1, and mucin synthesis, regardless of diet [142,143]. 

A proposed mechanism links intestinal dysbiosis, obesity, and metainflammation, and identifies the intestinal barrier as the trigger of an “inflammasome–microbiota axis” [144]. In this model, dysbiosis promotes a chronic low-grade inflammation that culminates in the release of pro-inflammatory cytokines, affecting metabolic, immune, and hepatic homeostasis [137]. 

## 4. The Gut–Liver Axis

The gut and liver are anatomically and functionally connected, forming the so-called “gut–liver axis”. This strict relationship is realized by anatomical structures (i.e., the biliary tract, portal vein system) and circulating products deriving from the immune system and gut microbiota [145,146,147,148]. As previously discussed, MAMPs and PAMPs derived from the gut reach the liver through the portal system and the systemic circulation through mesenteric lymph nodes, binding to TLRs and leading to the activation of inflammatory pathways. Bacterial translocation, even if physiological within certain limits, can be the original factor triggering liver injury or an additional cause in patients with other pre-existing viral or metabolic diseases and increased intestinal permeability, in particular those with liver cirrhosis. 

### 4.1. Liver Cirrhosis

**Figure 2:**
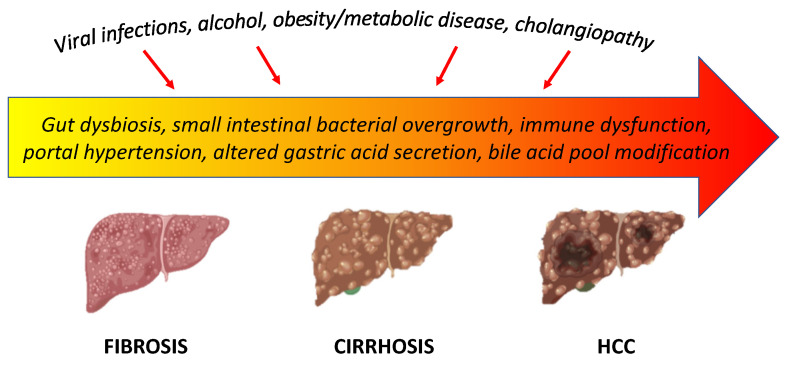
Gut–liver axis in the evolution of liver cirrhosis.

Dysbiosis, impairment of the intestinal barrier, and activation of alterations of the immune system play an important role in the evolution of chronic liver disease. Dysbiosis, small intestinal bacterial overgrowth, intestinal dysfunction due to portal hypertension, impaired immune response, and altered gastric acid and bile acid secretion are the most recognized factors involved in the intestinal barrier imbalance in patients with liver cirrhosis, leading to bacterial translocation (Figure 2) [149].

The reduction in autochthonous protective bacteria, such as Lachnospiraceae and Ruminococcaceae, and the increase in pathogens, such as Staphylococcaeae, Enterobacteriaceae, and Enterococcaceae, are the hallmark of dysbiosis in patients with liver cirrhosis. Intercurrent events such as infections or hepatic decompensation are associated with major changes in the gut microbiota [150]. Changes in the gut microbiota profile are observed in any tract of the gastrointestinal system, including the oral mucosa [151]. Translocation to the gut of oral pathogens capable of degrading intestinal mucus has recently been demonstrated in cirrhotic patients with hepatic encephalopathy, suggesting that the oral-gut–liver axis is involved in the pathogenesis of liver disease complications [152]. 

Recent studies on mice with defective leptin pathways show how obese mutant mice are featured by major defects in bile acid synthesis and conjugation, together with higher expression of liver cholic acid [142,153]. 

The end result of these alterations is that the gut microbiota is qualitatively and quantitively altered and bacterial translocation exacerbated in cirrhotic patients, fostering a pro-inflammatory response [30]. A large amount of PAMPs reaches the liver through the portal circulation, and is recognized by TLRs, such as TLR4 on the surface of Kupffer cells, endothelial cells, and stellate cells, triggering a pro-inflammatory response [154]. The TLR4-Myd88-NF-kB cascade culminates is the activation of hepatic stellate cells, resulting in damage and fibrogenesis through TGF-β signaling by Kupffer cells [155]. LPS stimulation of nitric oxide synthase (iNOS) further worsens portal hypertension, endothelial dysfunction, and liver toxicity [156,157,158]. Impairment of BA metabolism is also involved in the pro-inflammatory cascade of cirrhosis. Indeed, liver disease is associated with impaired BA metabolism, in particular a lower fecal BA concentration due to decreased synthesis, and a reduced primary to secondary bile acid conversion due to dysbiosis [159,160]. BAs interact with the farnesoid x receptor (FXR), a nuclear receptor present in the liver as well as in the gut, involved in the regulation of BA synthesis, glucose and lipid metabolism, and inflammatory response [161,162,163,164]. The modification of the BA pool in cirrhotic patients and the consequent dysfunction of FXR lead to a pro-inflammatory response [146].

Inflammation worsens liver damage and promotes the evolution of cirrhosis and its complications, including hepatocellular carcinoma (HCC) [165].

### 4.2. Non-Alcoholic Fatty Liver Disease (NAFLD)

Non-alcoholic fatty liver disease (NAFLD) is associated with chronic immune activation and adipose tissue inflammation, which have been linked with endotoxemia [166]. The connection between diet, gut microbiota imbalance, and metabolic disorders has been confirmed by the evidence that an HFD, western diet, and high-sugar diet can increase serum LPS concentration in mice, and subcutaneous infusion of LPS can induce insulin resistance in the liver [83]. Animals fed an HFD are more prone to develop non-alcoholic steatohepatitis (NASH) and steatosis when they are subjected to deletion of F11r, a gene that encodes for the TJ junctional adhesion molecule A (JAM-A) protein. Deletion of F11r is associated with increased intestinal inflammation and mucosal permeability, as confirmed by increased LPS serum levels [167]; in addition, TLR4 mRNA expression increases in mice Kupffer cells during HFD, causing oxidative stress in hepatocytes and leading to NASH [168]. In contrast, antibiotic treatment with polymyxin B improves hepatic steatosis in mice fed by total parenteral nutrition, by reducing the abundance of Gram-negative gut bacteria [169].

Dietary fructose, commonly found in sweetened beverages or as a food additive, is associated with metabolic syndrome and diabetes, increased hepatic lipogenesis, and steatosis [170,171]. Adolescents with NAFLD have higher LPS serum levels than healthy controls after fructose consumption [172]. In a rat model, the effect of fructose on intestinal permeability was linked to the activation of the LPS-TLR4-NF-kB pathway, resulting in the activation of Kupffer-cells and impaired TJ expression in the small intestine [173]. 

Recently, NASH has been associated with the disruption of the gut vascular barrier (GVB) [174]. Mouries et al. found evidence of intestinal barrier and GVB disruption in an experimental mouse model of NASH recreated by HFD administration. The expression of plasmalemmal vesicle-associated protein-1 (PV-1), a glycoprotein involved in endothelial permeability and dysfunction [175], increased 1 week after HFD initiation and was associated with bacterial translocation to the liver, development of insulin resistance, and NASH. When the fecal microbiota of HFD mice was transplanted into recipient mice, GVB damage was recapitulated. This demonstrates that both gut epithelial and vascular barrier damage are necessary for the development of NASH and enhance the role of gut dysbiosis in this process [176]. 

Patients with NASH show a high prevalence of small intestinal bacterial overgrowth (SIBO), and several gut microbial signatures associated with fibrosis have been proposed. Patients with more severe histological damage have an increased abundance of *Escherichia* spp., *Proteobacteria*, *Enterobacteriaceae*, *Ruminococcus*, and *Bacteroides* [177,178]. Compared with healthy people, the gut microbiota of NASH-related cirrhotic patients is characterized by the enrichment in *Veillonella parvula*, *Veillonella atypica*, *Ruminococcus gnavus*, *Clostridium bolteae*, and *Acidaminococcus* sp. D21, and a depletion of *Eubacterium eligens*, *Eubacterium rectale*, and *Faecalibacterium prausnitzii* [179]. Loss of gut microbiota diversity is also typical of advanced liver disease [179,180]; recently, the blooming of Erysipelotrichales has been reported in a mouse model during disease development, with possible correlation to intestinal inflammation [181]. 

This pro-inflammatory shift in the composition of the gut microbiota, in association with increased intestinal permeability, is a key element in the promotion and progression of liver damage, and is involved in the development of HCC in patients with NAFLD-related cirrhosis [182]. Indeed, this persistent immune activation may eventually lead to immune cell exhaustion and a profound depression of antitumor surveillance [165].

As a final observation, the gut microbiota can also fuel inflammation and liver damage through its metabolites. *E. coli* and *K. pneumoniae* are able to ferment dietary carbohydrates into ethanol, which, upon reaching the liver through the portal circulation, causes oxidative stress and may promote the development of NASH. Histological damage is aggravated by concomitant HFD and ameliorated by antibiotics administration or weight loss, the latter associated with a marked reduction in blood ethanol concentration [183,184]. 

### 4.3. Alcoholic Liver Disease (ALD)

Similarly to other liver diseases, alcoholic liver disease (ALD) is associated with an intestinal barrier impairment, because both alcohol and its metabolites are toxic to the gut [185,186].

The microbiota profile in patients with ALD is characterized by an increase in pathogenic bacteria and a decrease in anti-inflammatory bacteria such as *Akkermansia* [187,188,189]. In patients with alcoholic hepatitis, the presence of cytolysin-positive *Enterococcus faecalis* in a stool sample was positively associated with worse outcome [190]. FMT from humans colonized by cytolisin-positive *Enterococcus faecalis* into recipient ethanol-fed mice resulted in the translocation of this bacterium into the liver and was associated with severe liver damage; at the same time, treatment with bacteriophages against the exotoxin was effective in reducing the extent of liver injury.

Ethanol-induced dysbiosis is associated with decreased bacterial production of indole-3-acetic acid (IAA) from tryptophan. IAA ligates aryl-hydrocarbon receptor (AHR) and induces IL-22 expression [191]. IL-22 plays a protective role in ALD [192], and is also able to increase the expression of C-type lectin REG3G, an antimicrobial protein that reduces bacterial translocation in experimental models of ALD [191]. Studies have documented that ethanol causes a downregulation of REG3G and REG3B in the small intestine [193] and that the liver is protected against alcoholic steatohepatitis in case of REG3G hyper-expression [194]. This reinforces the evidence that dysbiosis leads to ALD through intestinal bacterial translocation and systemic inflammation [195]. Indeed, patients show higher serum levels of LPS and pro-inflammatory cytokines than healthy controls [189].

Recent evidence shows that intestinal permeability in ALD may be regulated by the impairment not only of the intestinal epithelial barrier but also of the GVB [196]. Indeed, the expression of PV-1, the marker of GVB destruction, was increased in the small intestine of ethanol-fed mice, with partial improvement after *Akkermansia muciniphila* administration [197]. 

## 5. Therapeutic Interventions to Restore Intestinal Barrier Integrity

### 5.1. Pharmacological Treatments

Several agents have been proposed to enhance or restore the integrity of the intestinal barrier. Although no specific drug is currently approved, most of them show interesting mechanisms of action and a promising future role in the modulation of intestinal permeability [198]. 

Probiotics are defined as live microorganisms, which when consumed in adequate amounts, confer a health effect on the host [199]. They are present in fermented foods, naturally or added artificially, and some of them colonize the human gut. The main probiotic microorganisms studied are *Lactobacillus*, *Bifidobacterium* and *Saccharomyces*. Although their specific role is unknown, evidence shows that probiotics may participate in healing the intestinal epithelium in several ways [200]. For example, *Lactobacillus plantarum* MB452, can increase the expression of TJ genes in Caco-2 cells [201], while *Lactobacillus rhamnosus* GG (LGG) plays a central role in epithelial cell survival in response to pro-apoptotic signaling pathways through activation of Akt and inhibition of p38 [202].

Next-generation probiotics are a potential new class of therapeutic drugs consisting of microorganisms not previously used, such *Akkermansia*, *Bacteroides*, and *Faecalibacterium*, deriving from gut microbiota [203]. Multi-omics studies have selected several commensals with metabolic and anti-inflammatory properties that could act as targeted and individualized adjuvants in fighting chronic diseases. The production of this new class of probiotics is still challenging, but *Akkermansia* seems to be the most promising agent that will be available in the near future [204,205].

Prebiotics are selectively fermented ingredients that result in specific changes in the composition and/or activity of the gastrointestinal microbiota, thus conferring benefits upon host health [206]. They consist primarily of fructo-oligosaccharides (FOS) and galacto-oligosaccharides (GOS), which are fermented by gut bacteria into SCFAs [207]. *Eubacterium rectale*, *Clostridium coccoides*, and *Roseburia* are the main bacteria producing butyrate, a type of SCFA that provides nutrition to colonocytes [208]. SCFAs are associated with increased mucus production on the intestinal surface and with maintenance of immune homeostasis and induction of T-reg lymphocytes in mice [15,209]. Inuline, a type of prebiotic, may act as scavenger by reducing damage on human colonic cells induced by LPS exposure [210]. 

Glutamine is a non-essential amino acid which plays a protective role in maintaining the integrity of the intestinal barrier by enhancing the survival of intestinal cells during stressful events and their proliferation, through activation of specific protein kinases [211,212]. Glutamine has also been studied as a protective agent against acetaldehyde, an alcohol-derived carcinogen. Pretreatment with glutamine has been shown to be effective in reducing disassembly of occludin, ZO1, E-cadherin, and β-catenin in human colonic cells exposed to acetaldehyde [213]. Another role played by glutamine harnesses its ability to modulate inflammation by targeting the NF-kB pathway [214]. 

Vitamin D increases the expression of TJ proteins such as claudin and occludin [215]. It also regulates antimicrobial responses on the intestinal surface and improves gut dysbiosis [216]. In particular, vitamin D supplementation increases the microbiota diversity of the gut microbiota and decreases the Firmicutes/Bacteroidetes ratio [217]. For these reasons, it has been proposed as an adjuvant therapy for patients with IBD, SpA, and T1DM [218,219,220]. 

Metformin, a drug used for T2DM treatment, has recently been proposed as a potential treatment for intestinal leakiness. In a mouse model, metformin was able to reduce HFD-induced hepatic steatosis and adipocyte inflammation by reducing cytokine expression and increasing colonic mucus production [221]. 

Obeticholic acid (OCA) is a semi-synthetic hydrophobic analogue of bile acids and an agonist of the farnesoid X receptor (FXR), which regulates bile acid metabolism. OCA is approved for the treatment of primary biliary cholangitis [222]. Recently, OCA has been shown to restore GVB integrity and reduce bacterial translocation in experimental models [223,224]. 

Divertin is a novel molecule that acts on the peri-junctional actomyosin ring (PAMR) of intestinal cells. Divertin blocks the activity of the mitochondrial hydroxylase (MCLK1) on PAMR, thereby reducing actomyosin ring contraction and tightening intercellular junctions. Because of these favorable effects on intestinal permeability, it has been proposed as a treatment for IBDs and other autoimmune diseases [225]. 

### 5.2. Non-Pharmacological Treatments

Decaffeinated coffee consumption has been reported to reduce the expression of *TLR4 and* LPS-binding protein (LBP) expression in HFD-fed mice. One of the possible mechanisms proposed is a coffee-mediated reduction in intestinal permeability [226]. 

Hot spices (cayenne pepper, chili pepper, paprika) are able to increase intestinal permeability to ions and reduce TJ expression [227]. In contrast, ginger has been shown to reduce the severity of intestinal damage in a mouse model of DSS ulcerative colitis by modifying the inflammatory cytokine pathways and the composition of the gut microbiota [228]. 

Flavonoids are natural phenolic substances found in plants and associated with various health properties in humans [229]. Genistein, one of the major flavonoids in soy, is a potent inhibitor of protein kinases, and experimental models have demonstrated its ability to reduce acetaldehyde-induced TJ damage [230,231,232]. It was able to prevent TJ damage induced by *E. coli* in an experiment on broilers [233]. 

Finally, intestinal bacteria conversion of tryptophan to tryptamine and indole metabolites, such as IAA and indole aldehyde, plays a protective role in barrier function [234]. Indole acetic acid produced by Lactobacilli during infections leads to the release of IL-22, a cytokine that can enhance mucosal immunity in mice and restore epithelial integrity [43,235,236]. At the same time, the IL-22 pathway appears to have metabolic benefits, too, improving insulin sensitivity and decreasing endotoxemia, preventing the development of metabolic syndrome [237].

## 6. Conclusions

**Figure 3:**
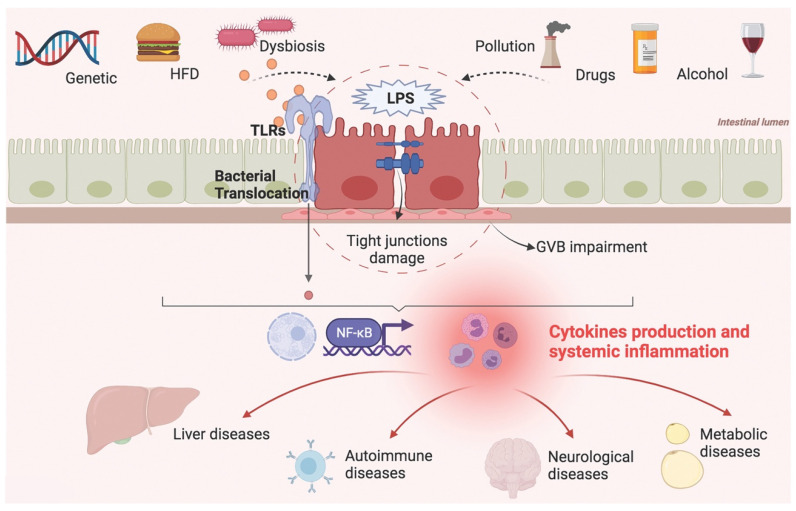
Factors contributing to intestinal barrier impairment and systemic diseases. HFD: high fat diet; LPS: lipopolysaccharide; GVB: gut vascular barrier; TLRs: toll-like receptors.

Targeting intestinal barrier homeostasis has become a challenge for the diagnosis and treatment of a large number of diseases. The composition and function of the gut microbiota and its pathophysiological role in human health have been active fields of research in recent decades. Lately, the attention of scientists has been drawn to the role of dysbiosis and intestinal barrier permeability in systemic diseases, elaborating the concept of a gut–liver axis, gut–brain axis, and gut–joint axis, but also gut–kidney axis, gut–eye axis, and so on. In these models, the gut represents the gateway through which external factors trigger systemic inflammation and tissue damage (Figure 3). 

Genetic susceptibility, environmental factors, dietary habits, and changes in the composition of the gut microbiota can affect the intestinal epithelial and vascular barrier, facilitating bacterial translocation and endotoxemia. These factors trigger a systemic inflammatory response that worsens organic and metabolic disorders.

The gut microbiota participates in this mechanism not only as a bystander but as an active player, modulating both positively and negatively the intestinal permeability through metabolic and immune pathways.

Despite the growing body of evidence supporting the etiologic link between intestinal permeability and several diseases, including extraintestinal ones, the exact mechanisms are still under investigation. Therefore, gut-focused therapeutic approaches that can modulate bacterial translocation and chronic inflammation are still in their preliminary stages, but are one of the most promising fields of research for development in the near future.

## Data Availability

No new data were created or analyzed in this study. Data sharing is not applicable to this article.
